# Supplementary material for: Remodeling of the Histoplasma Capsulatum Membrane Induced by Monoclonal Antibodies
Source: Vaccines (Basel). 2020 Jun 2;8(2):269. doi: 10.3390/vaccines8020269 (PMC7349930; doi:10.3390/vaccines8020269)
Supplement: Supplementary file 1 [file vaccines-08-00269-s001.zip › Burnet_Vaccines_SupplementalFigure_Rev1.pdf]

vaccines

MDPI

Supplemental information

Remodeling of the *Histoplasma capsulatum* membrane induced by monoclonal antibodies

Meagan C. Burnet, Daniel Zamith-Miranda, Heino M. Heyman, Karl K. Weitz, Erin L. Bredeweg, Joshua D. Nosanchuk and Ernesto S. Nakayasu

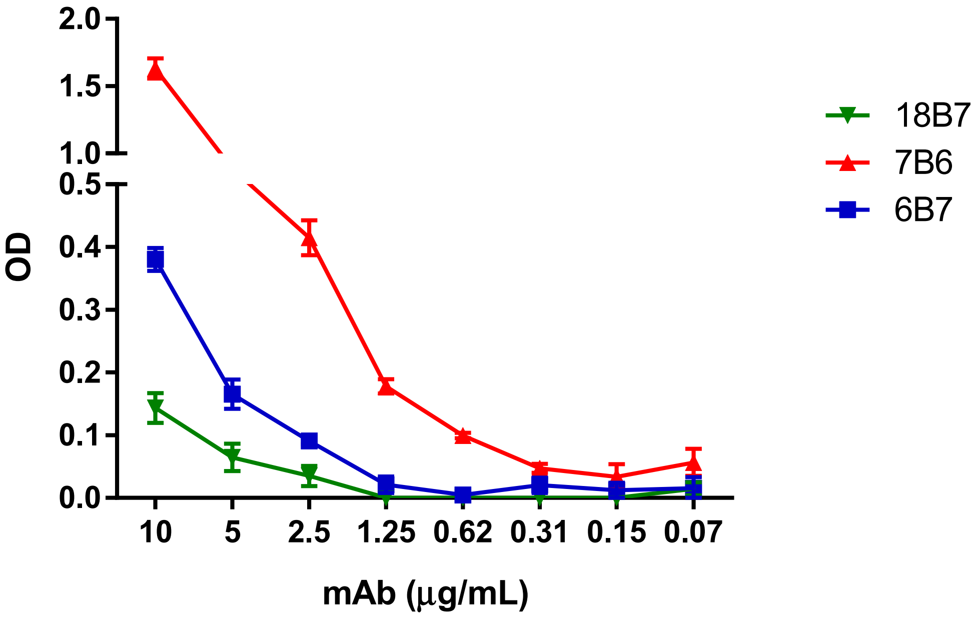

Figure S1. mAb binding to *H. capsulatum*. mAb binding was tested by ELISA against *H. capsulatum* yeast cells immobilized on the plate using serial dilutions of the mAb 18B7, 7B6 and 6B7.

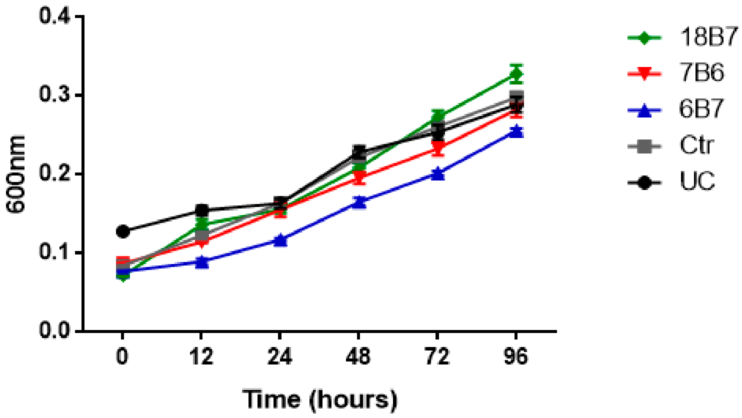

Figure S2. *Histoplasma capsulatum* growth in the presence of monoclonal antibodies. Cells were grown in Ham's F12 in the presence or absence of 6B7, 7B6 and 18B7 mAb (10 µg/mL). Growth was monitored by absorbance at 600 nm in a plate reader.
